# One-year survival in acute stroke patients requiring mechanical ventilation: a multicenter cohort study

**DOI:** 10.1186/s13613-020-00669-5

**Published:** 2020-05-07

**Authors:** Etienne de Montmollin, Nicolas Terzi, Claire Dupuis, Maité Garrouste-Orgeas, Daniel da Silva, Michaël Darmon, Virginie Laurent, Guillaume Thiéry, Johana Oziel, Guillaume Marcotte, Marc Gainnier, Shidasp Siami, Benjamin Sztrymf, Christophe Adrie, Jean Reignier, Stephane Ruckly, Romain Sonneville, Jean-François Timsit, OUTCOMEREA Study Group, Jean‐François Timsit, Elie Azoulay, Maïté Garrouste‐Orgeas, Jean‐Ralph Zahar, Christophe Adrie, Michael Darmon, Christophe Clec’h, Corinne Alberti, Adrien Français, Aurélien Vesin, Stephane Ruckly, Sébastien Bailly, Frederik Lecorre, Didier Nakache, Aurélien Van‐nieuwenhuyze, Romain Hernu, Carole Agasse, Bernard Allaouchiche, Pascal Andreu, Olivier Andremont, Laurent Argaud, Claire Ara‐Somohano, Elie Azoulay, Déborah Boyer, Jean‐Pierre Bedos, Thomas Baudry, Jérome Bedel, Julien Bohé, Lila Bouadma, Jeremy Bourenne, Noel Brule, Cédric Brétonnière, Christine Cheval, Julien Carvelli, Christophe Clec’h, Elisabeth Coupez, Michael Darmon, Etienne de Montmollin, Loa Dopeux, Anne‐Sylvie Dumenil, Claire Dupuis, Jean‐Marc Forel, Marc Gainnier, Charlotte Garret, Steven Grangé, Antoine Gros, Akim Haouache, Romain Hernu, Tarik Hissem, Vivien Hon Tua Ha, Sébastien Jochmans, Jean‐Baptiste Joffredo, Hatem Kallel, Guillaume Lacave, Alexandre Lautrette, Virgine Lemiale, Mathilde Lermuzeaux, Guillaume Marcotte, Jordane Lebut, Maxime Lugosi, Eric Magalhaes, Sibylle Merceron, Bruno Mourvillier, Benoît Misset, Bruno Mourvillier, Mathilde Neuville, Laurent Nicolet, Johanna Oziel, Laurent Papazian, Benjamin Planquette, Jean‐Pierre Quenot, Aguila Radjou, Marie Simon, Romain Sonneville, Jean Reignier, Bertrand Souweine, Carole Schwebel, Shidasp Siami, Roland Smonig, Gilles Troché, Marie Thuong, Guillaume Thierry, Dany Toledano, Guillaume Van Der Meersch, Marion Venot, Olivier Zambon

**Affiliations:** 1 Université de Paris, UMR 1137, IAME, Paris, France; 2 APHP, Medical and Infectious Diseases Intensive Care Unit, Bichat-Claude Bernard Hospital, 46 Rue Henri Huchard, 75018 Paris, France; 3 Medical Intensive Care Unit, Grenoble University Hospital, La Tronche, France; 4 Medical Intensive Care Unit, Gabriel Montpied University Hospital, Clermont-Ferrand, France; 5 Medical Unit, French British Hospital, Levallois-Perret, France; 6 Intensive Care Unit, Delafontaine Hospital, Saint-Denis, France; 7 Medical Intensive Care Unit, Saint-Louis Hospital, Paris, France; 8 Intensive Care Unit, André Mignot Hospital, Le Chesnay, France; 9 Intensive Care Unit, Hôpital Nord, Saint-Etienne, France; 10 APHP, Intensive Care Unit, Avicenne Hospital, Bobigny, France; 11 Surgical Intensive Care Unit, Lyon, France; 12 Intensive Care Unit, La Timone Hospital, Marseille, France; 13 Intensive Care Unit, Sud-Essonne Hospital, Etampes, France; 14 APHP, Intensive Care Unit, Antoine Béclère Hospital, Clamart, France; 15 APHP, Physiology Department, Cochin Hospital, Paris, France; 16 Medical Intensive Care Unit, Nantes University Hospital, Nantes, France; 17 Université de Paris, UMR 1148, LVTS, Paris, France

**Keywords:** Ischemic stroke, Intracranial hemorrhage, Subarachnoid hemorrhage, Intensive care, Mechanical ventilation, Endotracheal intubation

## Abstract

**Background:**

Most prognostic studies in acute stroke patients requiring invasive mechanical ventilation are outdated and have limitations such as single-center retrospective designs. We aimed to study the association of ICU admission factors, including the reason for intubation, with 1-year survival of acute stroke patients requiring mechanical ventilation.

**Methods:**

We conducted a secondary data use analysis of a prospective multicenter database (14 ICUs) between 1997 and 2016 on consecutive ICU stroke patients requiring mechanical ventilation at admission. We excluded patients with stroke of traumatic origin, subdural hematoma or cerebral venous thrombosis. The primary outcome was survival 1 year after ICU admission. Factors associated with the primary outcome were identified using a multivariable Cox model stratified on inclusion center.

**Results:**

We identified 419 patients (age 68 [58–76] years, males 60%) with a Glasgow coma score (GCS) of 4 [3–8] at admission. Stroke subtypes were acute ischemic stroke (AIS, 46%), intracranial hemorrhage (ICH, 42%) and subarachnoid hemorrhage (SAH, 12%). At 1 year, 96 (23%) patients were alive. Factors independently associated with decreased 1-year survival were ICH and SAH stroke subtypes, a lower GCS score at admission, a higher non-neurological SOFA score. Conversely, patients receiving acute-phase therapy had improved 1-year survival. Intubation for acute respiratory failure or coma was associated with comparable survival hazard ratios, whereas intubation for seizure was not associated with a worse prognosis than for elective procedure. Survival did not improve over the study period, but patients included in the most recent period had more comorbidities and presented higher severity scores at admission.

**Conclusions:**

In acute stroke patients requiring mechanical ventilation, the reason for intubation and the opportunity to receive acute-phase stroke therapy were independently associated with 1-year survival. These variables could assist in the decision process regarding the initiation of mechanical ventilation in acute stroke patients.

## Background

Stroke represents one of the leading causes of mortality and disability worldwide, with important social and economic consequences [1]. Despite a decrease in mortality and disability-adjusted life-years over the last 20 years, mediated by improvement in general ICU care, development of stroke units [2] and effective reperfusion strategies in acute ischemic stroke [3, 4], the burden of stroke is likely to remain high.

During the acute phase of stroke, patients may require intensive care for various reasons, including altered mental status, seizures, medical complications (i.e., pneumonia, sepsis, hyponatremia) and for monitoring after neuroradiological or surgical procedures [5–7]. Large multicenter population studies show that mechanical ventilation (MV) for acute stroke is required in 10–15% of patients admitted to a hospital and is dependent on stroke subtype, being 3 to 4 times more frequent for subarachnoid hemorrhage (SAH) and intracranial hemorrhage (ICH) patients (i.e., 29 and 30% of cases), as compared to acute ischemic stroke (AIS) patients (i.e., 8% of cases) [8]. Prognosis of mechanically ventilated stroke patients appears to be poor, hospital mortality ranging from 53 to 57% [8–10] and 1-year mortality ranging from 60 to 92% [11–15]. The need for MV appears to be a major predictor of mortality, with a hazard ratio (HR) of 5.6 for 30-day mortality in 31,300 ischemic stroke patients from the United States [16]. Similarly, in another population-based study of 798,255 acute stroke patients, the need for MV reduced the probability of being discharged home from 37 to 12% [8]. Although the need for mechanical ventilation is used as a surrogate marker for clinical severity, the reason for endotracheal intubation may be associated with potentially rapidly reversible conditions (e.g., status epilepticus, pneumonia, sepsis or hydrocephalus) that may be associated with more favorable outcomes [17].

Studies evaluating predictors of outcome in MV stroke patients have shown that age, consciousness impairment, absence of brainstem reflexes, and infarct/hematoma volume are associated with impaired survival [10–13, 15, 18, 19]. However, most of these studies take place before the year 2000 while the intensive care management of acute stroke patients has rapidly evolved [20], and none of the studies conducted after 2000 report long-term survival [8–10]. Furthermore, most of these studies all have limitations to some extent, including single-center, retrospective designs with a small number of patients.

Thus, we aimed to study the association of intensive care unit (ICU) admission factors, including the reason for intubation, with survival 1 year after ICU admission in acute stroke patients requiring mechanical ventilation. We also sought to describe the evolution of patients’ characteristics and survival rates over the 20 years of the study period.

## Methods

### Patient data source

This study was conducted using data from the French prospective multicenter (*n* = 30 ICUs) OUTCOMEREA database, from patients included between 1996 and 2016. The OUTCOMEREA database has been described in previous publications and has been approved by the French Advisory Committee for Data Processing in Health Research (CCTIRS) and the French Informatics and Liberty Commission (CNIL, registration no. 8999262) [21, 22]. The database protocol was submitted to the Institutional Review Board of the Clermont-Ferrand University hospital (Clermont-Ferrand, France), who waived the need for informed consent (IRB no. 5891).

### Study population and definitions

We included adult patients with acute stroke, admitted to the intensive care unit (ICU) and requiring invasive mechanical ventilation within 24 h of ICU admission. All ICU stays in the database were screened for a diagnosis of stroke, using the International Classification of Diseases 10th Revision (ICD-10) codes I60 (“Subarachnoid hemorrhage”), I61 (“Intracerebral hemorrhage”), I62 (“Other nontraumatic intracranial hemorrhage”), I63 (“Cerebral infarction”) and I64 (“Stroke, not specified as hemorrhage or infarction”). ICU stays were considered as related to acute stroke in cases of (1) direct ICU admission following stroke onset, or (2) ICU admission during the initial acute care hospital stay following stroke onset. We excluded patients without hospitalization reports, and if the stroke was related to traumatic brain injury. The severity of illness was graded at ICU admission with the use of the Simplified Acute Physiology Score (SAPS II) [23] and the sequential organ failure assessment (SOFA) score [24]. Coma was defined as a Glasgow coma score (GCS) < 8 [25]. The non-neurologic SOFA was defined as the SOFA score without the neurologic component. Functional status at ICU discharge was graded retrospectively using the modified Rankin Scale (mRS) [26], using a simplified questionnaire based on medical charts [27]. For the analysis of temporal trends in patients’ outcomes, the study period was arbitrarily divided into 7-year periods: 1996–2002, 2003–2009 and 2010–2016.

### Data collection

Data were prospectively collected at admission (demographics, chronic disease, admission features, baseline severity indexes, admission diagnosis, and admission type), and daily throughout the ICU stay (clinical and biological parameters, assessment of organ functions, requirement for MV, length of stay (LOS), WLST decision, and vital status at ICU and hospital discharge), through an anonymized electronic case report form using the Vigirea, Rhea, and e-Rhea softwares (OutcomeRea, Aulnay-sous-Bois, France). Long-term survival after hospital discharge was collected by each local investigator. For each stay, we collected the following retrospective data in the medical charts: (1) stroke history, including date of stroke, location, acute-phase stroke therapy (i.e., thrombolysis or endovascular thrombectomy for AIS and neurosurgery or embolization for ICH and SAH); (2) chronic diseases potentially related to stroke, including arterial hypertension, atrial fibrillation, history of ischemic or hemorrhagic stroke, diabetes, chronic alcohol consumption and the mRS at ICU discharge [7].

### Statistical analysis

Quantitative variables are presented as median, 1st and 3rd quartiles, and compared between groups with the Wilcoxon test. Qualitative variables are presented as frequency and corresponding percentage and compared with the Chi-square test or Fisher exact test as appropriate. The primary outcome was long-term survival, assessed by survival 1-year after ICU admission. We considered that determinants of 1-year survival were not affected by competing risks, and we identified variables associated with 1-year survival using a Cox proportional hazard model stratified on inclusion center, with a backward selection procedure (threshold of 0.05). Variables entered in the model were non-collinear factors associated (*p *< 0.1) with the outcome of interest in univariate analysis. We also entered in the model clinically pertinent factors associated with stroke survival in the literature. For stratification, centers with less than 10% of the cohort were regrouped in one stratum. Missing data were all completely at random with less than 10% missing value per variable, and were handled by simple imputation with median/most frequent method [28]. All statistical analyses were carried out with SAS 9.4 (SAS Institute Inc., Cary, NC, USA). A *p* value of 0.05 and lower was considered statistically significant.

## Results

### Patients

Among 22106 ICU admissions from 30 French ICUs over the study period, we identified 419 stays corresponding to 419 unique patients from 14 ICUs, involving acute stroke and where mechanical ventilation was initiated within 24 h of admission (Additional file 1). Hospitals in which patients were admitted were academic in 232 (55%) cases, had a stroke unit in 383 (91%) cases, and had a neurosurgery unit and interventional radiology in 188 (45%) cases. ICUs in which patients were admitted were medical, polyvalent or surgical in 212 (51%), 201 (48%), and 6 (1%) cases, respectively. In 264 (63%) patients, the ICU was authorized for organ donation after brain death. The characteristics of each participating center are detailed in Additional file 2. The number of patients admitted throughout the 21 years of the study period varied, 34 (8%) being admitted from 1996 to 2002, 228 (54%) from 2003 to 2009, and 157 (37%) from 2010 to 2016. At 1 year, 25 (6%) patients were lost to follow-up and censored after a median delay of 46 [23; 92] days. The baseline characteristics of patients are presented in Table 1. Patients were predominantly males (60%), aged 68.2 [57.9; 76.3] years, with strokes classified as AIS, ICH and SAH in 191 (46%), 178 (43%) and 50 (12%) of cases, respectively. The main reasons for endotracheal intubation were altered mental status (72%), acute respiratory failure (12%) and seizures (8%).

**Table 1:** Baseline characteristics and their univariate association with 1-year survival tested by Cox proportional hazard model

Variable *N* (%) or median [Q1; Q3]	All *N* = 419	1-year survival			
		Alive *N* = 114	Dead *N* = 305	HR (95% CI)	*p*
Demographics/history					
Age, years	68.2 [57.9; 76.3]	67.2 [57.4; 74.8]	69.1 [58.4; 76.9]	1.00 (0.99; 1.00)	0.35
Male sex	251 (59.9)	66 (57.9)	185 (60.7)	1.01 (0.81; 1.27)	0.93
Hypertension	238 (57.3)	72 (63.2)	166 (55.1)	1.20 (0.95; 1.52)	0.12
Diabetes mellitus	81 (19.3)	21 (18.4)	60 (19.7)	1.05 (0.79; 1.39)	0.72
Atrial fibrillation/flutter	55 (13.3)	13 (11.4)	42 (14)	0.86 (0.63; 1.19)	0.38
BMI ≥ 30 kg/m^2^	62 (15.3)	15 (13.5)	47 (16)	0.91 (0.67; 1.25)	0.57
Charlson comorbidity index ≥ 1	215 (51.3)	63 (55.3)	152 (49.8)	1.16 (0.93; 1.45)	0.20
Stroke characteristics					
Stroke type					
Ischemic	191 (45.6)	66 (57.9)	125 (41)	Ref	< .01
Hemorrhagic	178 (42.5)	35 (30.7)	143 (46.9)	0.58 (0.45; 0.74)	.
SAH	50 (11.9)	13 (11.4)	37 (12.1)	0.59 (0.41; 0.85)	.
Acute-phase stroke therapy	70 (16.7)	33 (28.9)	37 (12.1)	1.96 (1.39; 2.78)	< .01
Time from stroke to ICU admission, days	1 [1, 2]	1.5 [1, 5]	1 [1, 2]	1.05 (1.02; 1.09)	< .01
ICU admission					
Period of admission					
1996–2002	34 (8.1)	9 (7.9)	25 (8.2)	Ref	0.55
2003–2009	228 (54.4)	64 (56.1)	164 (53.8)	0.83 (0.54; 1.25)	.
2010–2016	157 (37.5)	41 (36)	116 (38)	0.79 (0.51; 1.2)	.
Type of ICU admission					
Transfer from ward	150 (35.8)	56 (49.1)	94 (30.8)	Ref	< .01
Direct (from ED or home)	269 (64.2)	58 (50.9)	211 (69.2)	0.63 (0.5; 0.81)	.
Reason for intubation					
Elective procedure	12 (2.9)	10 (8.8)	2 (0.7)	Ref	< .01
Altered mental status	302 (72.1)	66 (57.9)	236 (77.4)	0.12 (0.03; 0.47)	.
Respiratory failure	52 (12.4)	19 (16.7)	33 (10.8)	0.19 (0.05; 0.79)	.
Seizure	34 (8.1)	19 (16.7)	15 (4.9)	0.30 (0.07; 1.33)	.
Cardiac arrest	19 (4.5)	0 (0)	19 (6.2)	0.04 (0.01; 0.17)	.
GCS at admission					
8–15	110 (26.3)	57 (50)	53 (17.4)	Ref	< .01
3–7	309 (73.7)	57 (50)	252 (82.6)	0.36 (0.27; 0.49)	.
SAPS 2	58 [47; 72]	46.5 [39; 58]	62 [53; 75]	0.95 (0.95; 0.96)	< .01
Non-neurologic SOFA	4 [1, 6]	3 [1, 6]	4 [2, 6]	1.03 (0.99; 1.07)	0.14

HR, hazard ratio; CI, confidence interval; BMI, body max index; SAH, subarachnoid hemorrhage; ICU, intensive care unit; ED, emergency department; GCS, Glasgow coma scale; SAPS, simplified acute physiology score; SOFA, Sequential Organ Failure Assessment

Patients characteristics and outcomes according to stroke subtype are presented in Table 2. Time from stroke onset to intubation differed between AIS and hemorrhagic strokes (ICH and SAH): AIS patients were admitted to the ICU for intubation 2 [1, 4] days (vs 1 [1] for ICH and 1 [1] for SAH, *p* < 0.01), and were more frequently admitted from the ward than directly from home or the emergency department (53% vs 23% for ICH and 14% for SAH, *p* < 0.01). Furthermore, the reason for intubation differed between stroke types, AIS patients being more frequently intubated for acute respiratory (24% vs 3% for ICH and 0% for SAH, *p* < 0.01). 39 (20%) of the 191 AIS patients and 35 (15%) of the 228 patients with SAH or ICH received an acute-phase therapy.

**Table 2:** Patients characteristics and outcomes, according to stroke subtype

Variable *N* (%) or median [Q1; Q3]	AIS *n* = 191	ICH *n* = 178	SAH *n* = 50	*p*
Demographics/history				
Age, years	69.4 [61.1; 76.5]	67.7 [57.7; 76]	62.5 [54.3; 76]	0.10
Male sex	129 (67.5)	105 (59)	17 (34)	<.01
Charlson comorbidity index ≥ 1	115 (60.2)	85 (47.8)	15 (30)	<.01
Period of admission				0.67
1996–2002	18 (9.4)	12 (6.7)	4 (8)	.
2003–2009	108 (56.5)	95 (53.4)	25 (50)	.
2010–2016	65 (34)	71 (39.9)	21 (42)	.
Stroke characteristics				
AIS location				
Anterior circulation	117 (61.6)	–	–	
Posterior circulation	69 (36.3)	–	–	
ICH location			–	
Lobar	–	76 (45.5)	–	
Deep	–	50 (29.9)	–	
Infratentorial	–	21 (24.6)	–	
Acute-phase stroke therapy^a^	39 (20.4)	16 (9)	19 (38)	< .01
Intravenous thrombolysis	17 (8.9)	–	–	
Intra-arterial thrombolysis	10 (5.2)	–	–	
Endovascular thrombectomy	8 (4.2)	–	–	
Craniectomy	4 (2.1)	1 (0.6)	0	
External ventricular drainage	–	9 (5.1)	7 (14)	
Surgical hematoma evacuation	–	6 (3.4)	1 (2)	
Aneurysm surgical clipping	–	2 (1.12)	1 (2)	
Aneurysm endovascular coiling	–	0	5 (10)	
Time from stroke to ICU admission, days	2 [1, 4]	1 [1]	1 [1]	< .01
ICU admission				
Type of ICU admission				< .01
Direct (from ED or home)	89 (46.6)	137 (77)	43 (86)	.
Transfer from ward	102 (53.4)	41 (23)	7 (14)	.
Reason for intubation				< .01
Altered mental status	116 (60.7)	154 (86.5)	32 (64)	.
Respiratory failure	46 (24.1)	6 (3.4)	0 (0)	.
Seizure	19 (9.9)	11 (6.2)	4 (8)	.
Cardiac arrest	5 (2.6)	5 (2.8)	9 (18)	
Elective procedure	5 (2.6)	2 (1.1)	5 (10)	.
GCS at admission	6 [3, 10]	3 [3, 6]	3 [3, 7]	< .01
GCS < 8	120 (62.8)	151 (84.8)	38 (76)	
SAPS 2	56 [45; 67]	61 [51; 77]	59.5 [50; 72]	< .01
Non-neurologic SOFA	4 [2, 7]	4 [1, 5]	3 [2, 6]	0.14
ICU stay				
Duration of mechanical ventilation, days	5 [3, 12]	3 [2, 8]	3 [1, 6]	< .01
Vasopressor support	92 (48.2)	78 (43.8)	28 (56)	0.30
Renal replacement therapy	17 (8.9)	4 (2.2)	6 (12)	< .01
Withdrawal/withholding of care	77 (40.3)	66 (37.1)	15 (30)	0.40
ICU length of stay, days	7 [4, 15]	3 [2, 9]	3.5 [2, 8]	< .01
Hospital length of stay, days^b^	15 [6, 35]	5 [2, 15]	4 [2, 16]	< .01
Survival rates				
ICU survival	89 (46.6)	50 (28.1)	18 (36)	< .01
Hospital survival	71 (37.2)	43 (24.2)	15 (30)	0.03
1-year survival^c^	54 (30.2)	30 (17.3)	5 (11.9)	< .01

AIS, acute ischemic stroke; ICH, intracranial hemorrhage; SAH, subarachnoid hemorrhage; ICU, intensive care unit; ED, Emergency Department; GCS, Glasgow coma scale; SAPS, simplified acute physiology score; SOFA, Sequential Organ Failure Assessment

^a^A single patient could benefit from more than one type of acute-phase stroke therapy

^b^8 missing data

^c^25 missing data

During ICU stay, 198 (47%) patients required vasopressor support, and 27 (6%) renal replacement therapy. The duration of invasive mechanical ventilation was 4 [2, 9] days. A decision of WLST was made in 158 (38%) cases, with a delay of 4 [2, 8] days. ICU and hospital lengths of stay were 5 [2, 11] and 9 [3, 27] days, respectively. ICU, hospital and 1-year survival rates were 37%, 31%, and 23%, respectively. Causes of death in ICU were brain death (96/262, 37%), death without any WLST (23/262, 9%) and death following WLST (143/262, 55%). In patients alive at ICU discharge, 36/157 (23%) had an mRS ≤ 3 at ICU discharge. Having an mRS ≤ 3 at ICU discharge was associated with improved 1-year survival (*p* = 0.017 by the log-rank test) (Additional file 3). When considering hospital survivors only (*n* = 128), an mRS ≤ 3 at ICU discharge was associated with a shorter post-ICU stay (14 [9; 25.5] days vs 23 [9; 51] days, p = 0.07).

### Factors associated with 1-year survival

Univariate analysis of variables associated with 1-year survival is presented in Table 1. Age, sex, and comorbidities, defined by a Charlson comorbidity index ≥ 1 were not associated with 1-year survival. Similarly, the presence of a stroke unit on site, and the period of inclusion were not associated with 1-year survival.

**Fig. 1:**
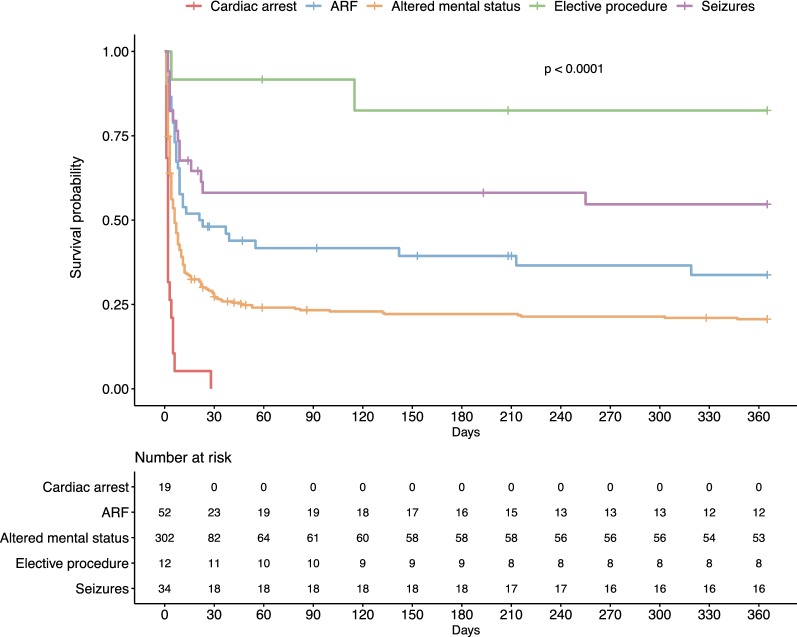
Kaplan–Meier’s survival estimates of patients according to the reason for endotracheal intubation

**Fig. 2:**
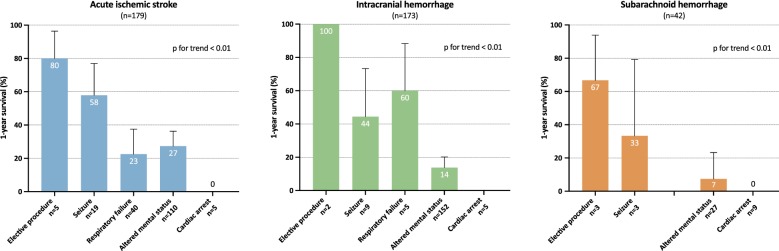
Survival rates according to stroke type and reason for endotracheal intubation

Multivariate analysis of variables associated with 1-year survival is presented in Table 3. Factors associated with decreased 1-year survival were ICH and SAH (compared to AIS), intubation for altered mental status or cardiac arrest (compared to intubation for an elective procedure), a GCS score < 8 and an increase in the non-neurologic SOFA score. In contrast, implementation of an acute-phase stroke therapy was the only variable associated with improved 1-year outcome. Kaplan–Meier’s survival estimates of patients according to the reason for endotracheal intubation are presented in Fig. 1. Survival rates according to stroke type and reason for endotracheal intubation are presented in Fig. 2 and show that the relation between the reason for endotracheal intubation and 1-year survival is consistent within all 3 stroke subtypes.

**Table 3:** Multivariable Cox proportional hazard model of factors associated with 1-year survival

Variable	HR	95% CI	*p*
Age > 70 years	0.8	[0.63; 1.01]	0.056
Male sex	0.96	[0.75; 1.23]	0.76
Stroke type			0.001
Ischemic	Ref		
Hemorrhagic	0.65	[0.51; 0.85]	
SAH	0.54	[0.36; 0.82]	
Reason for intubation			< 0.001
Elective procedure	Ref		
Seizure	0.55	[0.12; 2.53]	
Respiratory failure	0.22	[0.05; 0.95]	
Altered mental status	0.19	[0.04; 0.80]	
Cardiac arrest	0.08	[0.02; 0.38]	
GCS at ICU admission			< 0.001
8–15	Ref		
3–7	0.46	[0.34; 0.64]	
Non-neurologic SOFA, per point	0.95	[0.91; 0.99]	0.013
Acute-phase stroke therapy	1.81	[1.26; 2.60]	0.001

Variables entered in the model were age, gender, history of hemorrhagic stroke, medical vs surgical patient, type of ICU admission (ward vs home or emergency department), stroke type, acute-phase stroke therapy, reason for intubation, GCS at ICU admission, non-neurologic SOFA and were then selected using a backward procedure with a threshold of *p* = 0.05. Age and sex were considered clinically important factors and were forced into the model.

For stratification, centers with less than 10% of the cohort were regrouped in one stratum.

### Temporal trends in patients’ characteristics and 1-year survival

Characteristics of patients according to the period of inclusion are presented in the Additional file 4. Despite having similar survival rates at 1 year and a similar stroke type repartition, patients included in the most recent period (2010–2016) had more comorbidities (Charlson comorbidity index ≥ 1: 26% vs 49% vs 61%, *p* < 0.01) and presented higher admission SOFA scores (7 [6, 9] vs 7 [4, 9] vs 8 [5, 10], *p* = 0.02). One-year survival rates, GCS and stroke type repartition according to the period of inclusion are presented in the Additional file 5. During ICU stay, more patients needed vasopressor support (47% vs 41% vs 57%, *p* < 0.01) and renal replacement therapy (9% vs 4% vs 10%, *p* = 0.03). Over the 3 study periods, the duration of mechanical ventilation decreased significantly, as well as ICU length of stay and hospital length of stay. When considering only survivors (*n* = 157), duration of mechanical ventilation was not significantly different, but hospital length of stay significantly decreased over time.

## Discussion

In this reanalysis of a prospective database of 419 critically ill stroke patients requiring invasive mechanical ventilation, we show that survival 1 year after ICU admission is poor (23%) with no improvement over the 21 years of the study period. After adjustment for stroke subtype, neurological presentation and the extent of non-neurological organ failure, the reason for intubation remained independently associated with survival. Intubation for acute respiratory failure or coma was associated with comparable survival hazard ratios, whereas intubation for seizure was not associated with a worse prognosis than for elective procedure. By contrast, receiving an acute-phase therapy was associated with improved survival. Although 1-year survival did not improve over the study period, stroke patients included in the most recent period had more comorbidities and presented higher ICU admission SOFA scores.

The 1-year survival rate of 23% we found is consistent with previously published rates of 8-40% in studies focusing on MV stroke patients [11–15]. However, these studies have included patients admitted before the year 2000 and do not embrace the improvement in stroke care of the past 2 decades. To the best of our knowledge, there has been no study reporting long-term survival (i.e., at 1 year) in the specific population of MV stroke patients in the last 15 years. In our study, we show that from 1997 to 2016, with a stable stroke case mix over time, 1-year survival did not improve. This finding is surprising as the use of acute-phase stroke therapies increased over the study period (from 2.9% in 1996–2002 to 21% in 2010–2016). However, patients admitted to the ICU in the third period appeared to have more comorbidities and had more organ failures than in the previous 2, suggesting a modification of ICU admission policy over time. Thus, we hypothesize that the expected gain in survival brought by acute stroke therapies has been mitigated by increased severity of admitted stroke patients. Despite this increase in patient severity, ICU and hospital length of stay decreased both in the whole population and in survivors. More recently, in a United States population-based study of 99,700 stroke patients with MV included from 2005 to 2011 [8], hospital survival was 44%, compared to 31% in our study. Those figures are difficult to compare, as the case-mix of stroke subtype and the distribution of reason for intubation may be different. Among the 14 centers of our study, 5 centers (representing 188 (45%) patients) had on-site 24/7 interventional radiology, and it is likely that the admission policy of other participating centers was not oriented on procedural patients. Thus, we can hypothesize that the proportion of patients deemed neurologically too severe to be eligible for acute-phase stroke therapy or out of the window of therapeutic opportunity was higher in our cohort than in the most recently studied cohorts [8–10].

Among the factors associated with 1-year survival identified in our study, the reason for intubation appears to be a strong predictor. We found that intubation for a cardiac arrest or an altered mental status is associated with worse 1-year survival compared to intubation for an elective procedure. In particular, it is striking to note that in our cohort, there were no survivors in patients admitted for cardiac arrest following stroke. By comparison, in 352 AIS patients with in-hospital cardiac arrests, 1-year mortality was 96% [29], and in 92 patients with out-of-hospital cardiac arrest caused by ICH or SAH, there were no patients with a favorable neurologic outcome [30]. By contrast, intubation for a seizure was not associated with impaired outcome. Only four studies have previously assessed the impact of the reason for intubation and have shown that acute respiratory failure and coma were associated with worse outcomes [12, 15, 19, 31]. The reason for intubation appears to be a simple bedside clinical element that can assist the decision of admission of an acutely ill stroke patient.

The strengths of our study include a multicenter population from a high-quality prospective database with a focus on a well-defined population of acute stroke patients requiring invasive mechanical ventilation. However, our study has also limitations. First, the OUTCOMEREA database has not been built specifically for stroke studies, and all data regarding stroke are retrospective, collected from hospitalization records. Hence, data on potentially useful scores for prognostication in this setting, such as the NIHSS scores, are lacking [6]. Furthermore, only long-term vital status was available, and evaluation of long-term functional outcomes with an appropriate tool (i.e., the modified Rankin scale) would have added valuable information. Second, the results of the study may lack generalizability as this is an exclusively French cohort including only medical and polyvalent ICUs and no specialized neuro-ICU. Furthermore, only 45% of the cohort was treated with on-site neurosurgery and interventional radiology, and it is possible that we selected a population with a high proportion of patients not eligible for acute-phase stroke therapy. Although moderate-quality evidence suggests that admission to a specialized NICU compared to a general ICU improves outcome of all stroke patients [32–34], organization of acute stroke care in France allows admission to NICU mainly for comatose ICH patients deemed to benefit from early surgery, or severe SAH patients requiring endovascular treatment for treatment of ruptured aneurysm and/or invasive intracranial pressure monitoring. Third, our cohort comprised 3 distinct stroke etiologies (AIS, ICH, and SAH) that have different admission characteristics, risk factors, brain damage pathophysiology, complications, treatments, and prognosis. However, the results of the multivariate model are adjusted on the type of stroke, and Fig. 2 shows that the prognostic impact of the reason for intubation appears consistent among stroke subtypes. Fourth, as endovascular thrombectomy has mainly been developed after 2015, only a small fraction of our cohort is concerned and the survival trends we show may not take into account the recent survival benefits related to this procedure [4]. Fifth, as for all studies focusing on populations with a high rate of WLST, our study bears an inherent bias by self-fulfilling prophecy [35]. Sixth, as all centers did not participate throughout the 21 years of the study period, we cannot analyze any variation of incidence of admission of stroke patients with mechanical ventilation.

## Conclusions

In this secondary data use of a prospective multicenter cohort study of critically ill stroke patients requiring invasive mechanical ventilation, we show that 1-year survival is 23%, with no improvement over the 21 years of the study period, although admitted patient’s severity increased in the most recent period. The reason for intubation and the opportunity to receive an acute-phase stroke therapy were independently associated with long-term survival. These variables could assist the decision process regarding ICU admission and initiation of MV in acute stroke patients.

## Supplementary information

**Additional file 1.** Study flow diagram.

**Additional file 2.** Characteristics of inclusion centers.

**Additional file 3.** Patients characteristics and outcomes, according to the type of stroke.

**Additional file 4.** Kaplan–Meier’s survival estimates of ICU survivors according to the mRS at ICU discharge.

**Additional file 5.** Stroke type, ICU admission Glasgow Coma Score and 1-year survival rates, according to inclusion period.

## Data Availability

The datasets used and/or analyzed during the current study are available from the corresponding author on reasonable request.

## Abbreviations

AIS: Acute ischemic stroke
BMI: Body mass index
CI: Confidence interval
ED: Emergency department
GCS: Glasgow coma score
HR: Hazard ratio
ICH: Intracranial hemorrhage
ICU: Intensive care unit
LOS: Length of stay
mRS: Modified Rankin Scale
MV: Mechanical ventilation
NIHSS: National Institute of Health Stroke Scale
SAH: Subarachnoid hemorrhage
SAPS: Simplified Acute Physiology Score
SOFA: Sequential Organ Failure Assessment
WLST: Withhold/withdraw of Life-Sustaining Treatments
