# Supplementary material for: One-year survival in acute stroke patients requiring mechanical ventilation: a multicenter cohort study
Source: Ann Intensive Care. 2020 May 7;10:53. doi: 10.1186/s13613-020-00669-5 (PMC7205929; doi:10.1186/s13613-020-00669-5)
Supplement: Supplementary file 1 — Additional file 1. Study flow diagram. [file 13613_2020_669_MOESM1_ESM.docx]

**Additional files**

**One-year survival in acute stroke patients requiring mechanical ventilation: a multicenter cohort study**

**Author names and affiliations**

Etienne de Montmollin; Nicolas Terzi; Claire Dupuis; Maité Garrouste-Orgeas; Daniel da Silva; Michaël Darmon; Virginie Laurent; Guillaume Thiéry; Johana Oziel; Guillaume Marcotte; Marc Gainnier; Shidasp Siami; Benjamin Sztrymf; Christophe Adrie; Jean Reignier; Stephane Ruckly; Romain Sonneville; and Jean-François Timsit for the OUTCOMEREA Study Group

### **Additional file 1.** Study flow diagram
