# Supplementary material for: One-year survival in acute stroke patients requiring mechanical ventilation: a multicenter cohort study
Source: Ann Intensive Care. 2020 May 7;10:53. doi: 10.1186/s13613-020-00669-5 (PMC7205929; doi:10.1186/s13613-020-00669-5)
Supplement: Supplementary file 2 — Additional file 2. Characteristics of inclusion centers. [file 13613_2020_669_MOESM2_ESM.docx]

**Additional files**

**One-year survival in acute stroke patients requiring mechanical ventilation: a multicenter cohort study**

**Author names and affiliations**

Etienne de Montmollin; Nicolas Terzi; Claire Dupuis; Maité Garrouste-Orgeas; Daniel da Silva; Michaël Darmon; Virginie Laurent; Guillaume Thiéry; Johana Oziel; Guillaume Marcotte; Marc Gainnier; Shidasp Siami; Benjamin Sztrymf; Christophe Adrie; Jean Reignier; Stephane Ruckly; Romain Sonneville; and Jean-François Timsit for the OUTCOMEREA Study Group

### **Additional file 2.** Characteristics of inclusion centers

| **Centre** | **N (%)**  **included** | **Period of participation in the database** | **Hospital characteristics** | | | |  | **ICU characteristic** | | |
| --- | --- | --- | --- | --- | --- | --- | --- | --- | --- | --- |
|  |  |  | **Hospital type** | **Number**  **of beds** | **Stroke unit** | **Neurosurgery** | **Interventional radiology** | **ICU type** | **Number**  **of beds** | **Authorization for**  **organ donation** |
| 1 | 166 (39.6) | 2004-2013 | Academic | 2133 | Yes | Yes | Yes | Medical | 18 | Yes |
| 2 | 129 (30.8) | 1996-2016 | Non-academic | 584 | Yes | No | No | Polyvalent | 10 | No |
| 3 | 43 (10.3) | 2000-2008 | Non-academic | 400 | Yes | No | No | Polyvalent | 13 | Yes |
| 4 | 23 (5.5) | 1998-2014 | Academic | 540 | No | No | No | Medical | 12 | No |
| 5 | 12 (2.9) | 2010-2016 | Non-academic | 480 | Yes | No | No | Polyvalent | 17 | Yes |
| 6 | 11 (2.6) | 2000-2004  2012-2016 | Academic | 945 | Yes | No | No | Medical | 20 | Yes |
| 7 | 11 (2.6) | 2010-2013 | Academic | 1919 | Yes | Yes | Yes | Medical | 15 | Yes |
| 8 | 9 (2.1) | 1997-2014 | Academic | 500 | No | No | No | Polyvalent | 16 | Yes |
| 9 | 6 (1.4) | 2006-2013 | Academic | 964 | Yes | Yes | Yes | Surgical | 20 | Yes |
| 10 | 4 (1) | 2015 | Academic | 1069 | Yes | Yes | Yes | Polyvalent | 10 | Yes |
| 11 | 2 (0.5) | 2002-2011 | Non-academic | 308 | No | No | No | Polyvalent | 10 | No |
| 12 | 1 (0.2) | 2016 | Non-academic | 450 | No | No | No | Polyvalent | 22 | Yes |
| 13 | 1 (0.2) | 2016 | Academic | 1500 | Yes | Yes | Yes | Medical | 20 | Yes |
| 14 | 1 (0.2) | 1997-2011 | Academic | 395 | No | No | No | Polyvalent | 12 | No |
