# Supplementary material for: One-year survival in acute stroke patients requiring mechanical ventilation: a multicenter cohort study
Source: Ann Intensive Care. 2020 May 7;10:53. doi: 10.1186/s13613-020-00669-5 (PMC7205929; doi:10.1186/s13613-020-00669-5)
Supplement: Supplementary file 4 — Additional file 4. Kaplan–Meier’s survival estimates of ICU survivors according to the mRS at ICU discharge. [file 13613_2020_669_MOESM4_ESM.docx]

**Additional files**

**One-year survival in acute stroke patients requiring mechanical ventilation: a multicenter cohort study**

**Author names and affiliations**

Etienne de Montmollin; Nicolas Terzi; Claire Dupuis; Maité Garrouste-Orgeas; Daniel da Silva; Michaël Darmon; Virginie Laurent; Guillaume Thiéry; Johana Oziel; Guillaume Marcotte; Marc Gainnier; Shidasp Siami; Benjamin Sztrymf; Christophe Adrie; Jean Reignier; Stephane Ruckly; Romain Sonneville; and Jean-François Timsit for the OUTCOMEREA Study Group

### **Additional file 4.** Patients characteristics and outcomes, according to inclusion period

| **Variable**  N (%) or median [Q1; Q3] | **1996-2002**  n=34 | **2003-2009**  n=228 | **2010-2016**  n=157 | **p** |
| --- | --- | --- | --- | --- |
| **Demographics/history** |  |  |  |  |
| Age, years | 68.1 [57.6; 74] | 68.5 [57.9; 75.7] | 68.2 [58.7; 77.8] | 0.44 |
| Male sex | 23 (67.6) | 126 (55.3) | 102 (65) | 0.10 |
| Charlson comorbidity index ≥ 1 | 9 (26.5) | 111 (48.7) | 95 (60.5) | <.01 |
| **Stroke characteristics** |  |  |  |  |
| Stroke type |  |  |  | 0.67 |
| SAH | 4 (11.8) | 25 (11) | 21 (13.4) | . |
| AIS | 18 (52.9) | 108 (47.4) | 65 (41.4) | . |
| ICH | 12 (35.3) | 95 (41.7) | 71 (45.2) | . |
| Acute phase stroke therapy | 1 (2.9) | 36 (15.8) | 33 (21) | 0.03 |
| Time from stroke to ICU admission, days | 1 [1; 2] | 1 [1; 2] | 1 [1; 2] | 0.65 |
| **ICU admission** |  |  |  |  |
| Type of ICU admission |  |  |  | 0.59 |
| Direct (from ED or home) | 24 (70.6) | 148 (64.9) | 97 (61.8) | . |
| Transfer from ward | 10 (29.4) | 80 (35.1) | 60 (38.2) | . |
| Reason for intubation |  |  |  | 0.54 |
| Elective procedure | 0 (0) | 5 (2.2) | 7 (4.5) | . |
| Altered mental status | 23 (67.6) | 172 (75.4) | 107 (68.2) | . |
| Respiratory failure | 5 (14.7) | 26 (11.4) | 21 (13.4) | . |
| Seizure | 4 (11.8) | 18 (7.9) | 12 (7.6) | . |
| Cardiac arrest | 2 (5.9) | 7 (3.1) | 10 (6.4) |  |
| GCS at admission | 6 [3; 7] | 4 [3; 8] | 3 [3; 7] | 0.22 |
| SAPS 2 | 57 [47; 67] | 56 [45; 72] | 60 [52; 73] | 0.07 |
| Non-neurologic SOFA | 4 [3; 5] | 3 [1; 6] | 4 [2; 7] | 0.02 |
| **ICU stay** |  |  |  |  |
| Duration of mechanical ventilation, days | 8 [4; 16] | 4 [2; 8] | 4 [2; 9] | <.01 |
| Duration of mechanical ventilation in survivors, days (n=157) | 8 [4 ; 15] | 6 [3 ; 15] | 5.5 [3 ; 12] | 0.71 |
| Vasopressor support | 16 (47.1) | 93 (40.8) | 89 (56.7) | <.01 |
| Renal replacement therapy | 3 (8.8) | 8 (3.5) | 16 (10.2) | 0.03 |
| WLST | 12 (35.3) | 86 (37.7) | 60 (38.2) | 0.95 |
| Time from ICU admission to WLST | 5 [2.5; 8.5] | 4 [2; 7] | 4 [2; 9] | 0.79 |
| ICU length of stay, days | 11.5 [4; 22] | 5.5 [2; 11] | 4 [2; 9] | <.01 |
| ICU length of stay, survivors only, days | 18 [8; 27] | 11 [5; 21] | 9 [5; 25] | 0.37 |
| Hospital length of stay, days † | 23.5 [9; 44] | 10 [3; 28] | 7 [3; 21] | <.01 |
| Hospital length of stay, survivors only, days † | 45 [26; 92] | 31 [15; 59] | 26.5 [15; 35] | 0.03 |
| **Survival rates** |  |  |  |  |
| ICU survival | 14 (41.2) | 87 (38.2) | 56 (35.7) | 0.79 |
| Hospital survival | 9 (26.5) | 71 (31.1) | 49 (31.2) | 0.85 |
| 1-year survival ‡ | 7 (21.9) | 49 (23) | 33 (22.1) | 0.98 |

Abbreviations: SAH, Subarachnoid Hemorrhage; AIS, Acute Ischemic Stroke; ICH, Intracranial hemorrhage; ICU, Intensive Care Unit; ED, Emergency Department; GCS, Glasgow Coma Scale; SAPS, Simplified Acute Physiology Score; SOFA, Sequential Organ Failure Assessment; WLST, Withdrawal or withholding of Life-Sustaining Treatment.

† 8 missing data, ‡ 25 missing data
